# Lateral herniation during treatment with collagenase Clostridium histolyticum (Xiaflex) for Peyronie’s disease

**DOI:** 10.1186/s12894-021-00858-9

**Published:** 2021-06-27

**Authors:** Margaret K. Gannon, Amy M. Pearlman

**Affiliations:** Department of Urology, Carver College of Medicine, University of Iowa, Iowa City, IA USA

**Keywords:** Xiaflex, Herniation, Peyronie’s disease, Collagenase, Collagenase Clostridium histolyticum

## Abstract

**Background:**

Collagenase Clostridium histolyticum (CCH), also know as Xiaflex, with penile modeling is considered to be the gold standard non-surgical option for management of Peyronie’s disease and is known to be safe and efficacious. Corporal rupture is a rare but known adverse event of CCH treatment, however there are limited studies describing corporal herniation without rupture. Here we present a patient who experienced a rare complication following CCH injections for Peyronie’s disease: lateral herniation of the tunica albuginea in the setting of a dorsal penile plaque.

**Case presentation:**

A 58-year-old male presented to our clinic seeking treatment for Peyronie’s disease. On exam, he was found to have a palpable dorsal plaque and > 30 degrees leftward curvature of the penis. He was deemed an appropriate candidate for and patient decided to proceed with CCH and modeling. He received 2 cycles of CCH injections (4 total CCH injections) with in-office and at-home penile modeling, per manufacturer’s protocol. Two weeks following in-office modeling during his second CCH cycle, the patient reported a painless, soft swelling involving the left side of his penile shaft only occurring with erection. Exam and history were suggestive of lateral herniation rather than corporal rupture. CCH was discontinued. Patient declined further evaluation with penile ultrasound.

**Conclusions:**

This is the first case report detailing lateral herniation with CCH injections. Symptoms and exam that should raise suspicion of corporal herniation are a soft, painless mass with erection.

## Background

Peyronie’s disease is a penile condition characterized by pain, curvature, and/or shortening of the penis and is often associated with erectile dysfunction and difficulty engaging in sexual intercourse [1]. Disorganized and excessive deposition of collagen likely results in the formation of a fibrous penile plaque within the tunica albuginea [2], most commonly observed in the dorsal midline [3].

Collagenase Clostridium histolyticum (CCH), also known as Xiaflex, injections with in-office and at-home penile modeling is an effective treatment option for reducing penile curvature for patients with Peyronie’s disease who meet specific inclusion criteria [4]. Common adverse events include penile hematoma formation (50.2%), pain (33.5%), and swelling (28.9%), however, these often resolve without intervention [1]. One of the most serious adverse events is corporal body rupture/penile fracture (0.9%) which may be related to modeling, resumption of sexual intercourse prior to recommended hiatus, or the injection itself [5].

Here, we present a patient who experienced a rarely reported complication following CCH injections for Peyronie’s disease: a lateral herniation following injection of a dorsal penile plaque.

## Case presentation

A 58-year-old Caucasian male with history of hyperlipidemia and recurrent nephrolithiasis presented to our clinic with treatment naive Peyroni’s disease. The patient reported a stable, leftward penile curvature that had been present for 3 years. The patient denied pain with erections or erectile dysfunction, however the curvature was affecting his ability to have intercourse. Using a goniometer to measure his curvature in the erect state, he was noted to have 20 and 32 degrees of leftward curvature at proximal one third and distal one third of penile shaft, respectively. A dorsal penile plaque was palpated approximately 2 cm distal to the penopubic junction. After discussing treatment options, the patient decided to proceed with CCH injections and penile modeling.

The patient received 0.58 mg intralesional CCH injection at the side of maximal curvature (the distal 1/3 of the penile shaft for this patient) on 2 occasions 1 day apart followed by in-office modeling 6 days after the second CCH injection, per protocol. He performed regular at-home penile modeling for 6 weeks. The patient then repeated the above injections, in-office modeling procedure, and daily at-home stretches, per protocol, for 2 total cycles. Other than transient ecchymosis, there were no complications or adverse events following either of the CCH cycles.

**Fig. 1:**
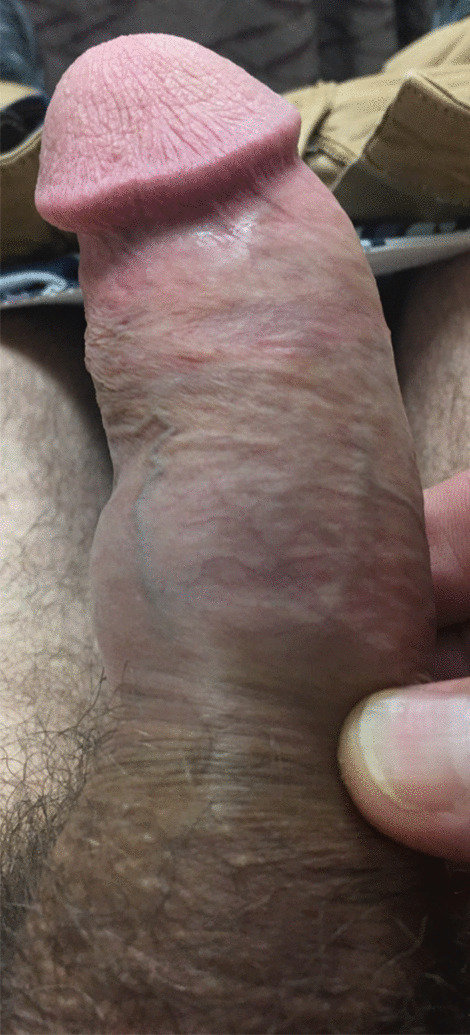
Lateral penile swelling following second cycle of Xiaflex/penile modeling; superior view

**Fig. 2:**
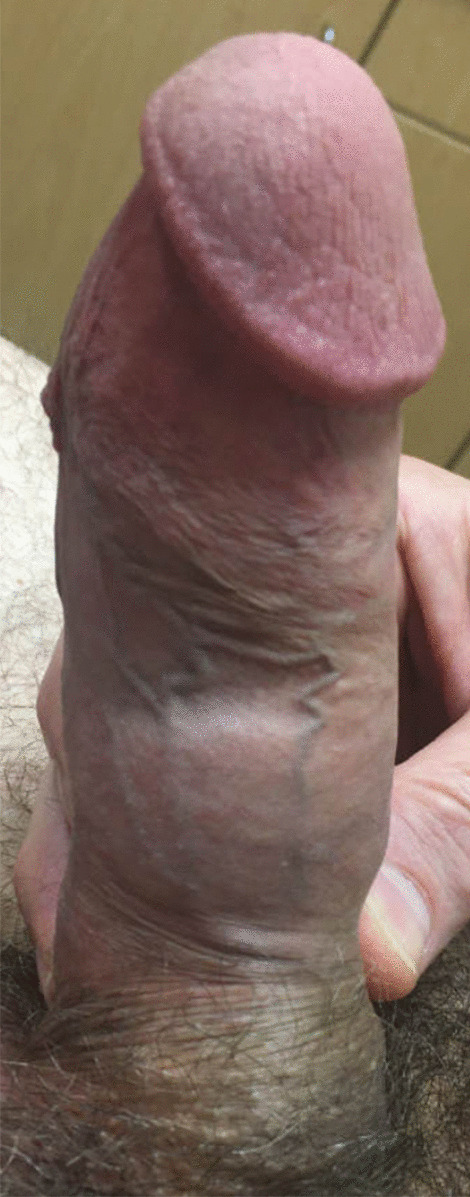
Lateral penile swelling following second cycle of Xiaflex/penile modeling; lateral view

The patient returned to clinic 6 weeks after completion of his second round of CCH injections to receive the first injection of his third cycle, however, he reported painless swelling on the left side of his penile shaft that he noticed 2 weeks after the second CCH injection of his second cycle. He denied engaging in intercourse, experiencing penile trauma or hearing any “popping” associated with the onset of the swelling. On exam, the flaccid penis did not have a palpable corporal defect, tunical thickening, or hematoma, and appeared grossly normal. Exam following a 20 mcg alprostadil injection showed a small amount of swelling was noted on the left side of the penile shaft distal to the base of the penile shaft (Figs. 1, 2). The swelling was soft and nontender without any overlying skin changes. The third cycle of CCH was deferred and the patient was instructed to wrap the penis in Coban and return to clinic in 1 week to reassess the swelling. At time of follow up, the patient reported continued outpouching of his left erectile body with erections. A repeat exam following a 20 mcg alprostdil injection was consistent with his previous exam. The patient declined further investigation of the swelling with Doppler ultrasound.

## Discussion and conclusions

This case illustrates a rarely reported complication of lateral herniation following CCH injections and penile modeling.

CCH with penile modeling is considered to be the gold standard non-surgical option for management of Peyronie’s disease [6]. The safety and efficacy of CCH has been demonstrated in large scale well-designed clinical trials [4, 5] and the adverse events of the treatment are well-known. Most patients (~ 93%) treated with CCH and modeling report at least one treatment associated adverse event, most commonly penile hematoma (≥ 25% of patients) [5]. Although most patients do experience treatment related adverse events, the vast majority are only mild or moderate in severity [5]. The minority of patients (~ 10%) who experience severe treatment related adverse events most commonly experience a severe penile hematoma (3.7%).

Corporal rupture or penile fracture is also a known treatment related serious adverse event of CCH and modeling. Large scale clinical trials have reported rates of less than 1%, although anecdotally there is controversy among experts about the prevalence of corporal rupture with CCH treatments. One study surveying providers reported a rupture rate of 34%, and found that most occurred after the second CCH cycle (28%) and 16% of those ruptures did not occur over the plaque/injection site [7].

The unique aspect of this case is that our patient’s adverse event described above was not consistent with a corporal rupture but rather lateral herniation, not well characterized in the existing literature. In fact, only one published case report has ever described this phenomenon [8]. Similar to our patient, this case report describes a painless, soft penile mass present only during erections. Doppler penile ultrasound confirmed the mass was vascular and upon surgical exploration, the patient was found to have local tunical attenuation and an aneurysmal dilation of the corpora. Tunica herniation was briefly mentioned in Brant et al. as a complication seen when injecting CCH into lateral penile plaques. In fact, the authors recommend avoiding CCH injections for lateral plaques in order to prevent herniation or microrupture of the tunica based on their anecdotal experience [9]. Although studies citing lateral herniation of the tunica following CCH are limited, it may be that this complication happens more than is currently appreciated in the current literature.

This case highlights a tunical herniation that did not occur at the site of the plaque or previous CCH injections. We can only speculate about the etiology of this complication. We do know that the lateral tunica thickness is less than that of the dorsal/ventral tunica (0.8 mm vs 2.2 mm), making it more susceptible to injury [10]. At-home modeling protocol involves stretching of the penis in its flaccid state, as well as straightening of the penis when erect, as per recommended protocol. It is possible that the patient may have caused trauma to the tunica during modeling though the patient denied any known occurrence of trauma. It is possible that the Peyronie’s disease complex of disordered collagen deposition and scar tissue formation had weakened the tunica in locations other than where a palpable plaque was formed. Alternatively, a CCH injection into healthy tissue or extravasation of the medication with manipulation of the needle in and out of the plaque could have compromised the surrounding tunica, although injections for our patient were localized to his plaque according to detailed procedure notes.

This is the first case report detailing lateral herniation following CCH injections and penile modeling for a palpable dorsal plaque. Symptoms of corporal herniation are a soft, painless mass with erection, and diagnosis can be confirmed with imaging or surgical exploration. Although this condition is rare, it is important to characterize such injuries so we can better understand how to prevent and manage them.

## Data Availability

Data sharing is not applicable to this article as no datasets were generated or analyzed during the current study.

## Abbreviation

CCH: Collagenase Clostridium histolyticum
